# Acquired von Willebrand syndrome in children with aorticand pulmonary stenosis

**DOI:** 10.5830/CVJA-2015-093

**Published:** 2016

**Authors:** Fatih Köksal Binnetoğlu, Kadir Babaoğlu, Şayegan Güven Filiz, Emine Zengin, Nazan Sarper, Gürkan Altun, Suar Çakı Kılıç

**Affiliations:** Department of Paediatric Cardiology, Çanakkale Onsekiz Mart University, Çanakkale, Turkey; Department of Paediatric Cardiology, Kocaeli University Medical Faculty, Kocaeli, Turkey; Department of Paediatrics, Karabük State Hospital, Karabük, Turkey; Department of Paediatric Haematology, Kocaeli University Medical Faculty, Kocaeli, Turkey; Department of Paediatric Haematology, Kocaeli University Medical Faculty, Kocaeli, Turkey; Department of Paediatric Cardiology, Kocaeli Derince Education and Research Hospital, Kocaeli Turkey; Department of Paediatric Haematology, Cumhuriyet University Medical Faculty, Sivas, Turkey

**Keywords:** aortic stenosis, children, pulmonary stenosis, von Willebrand

## Abstract

**Introduction:**

This prospective study was planned to investigate the frequency and relationship of acquired von Willebrand syndrome (AVWS) with aortic and pulmonary stenosis in patients.

**Methods:**

A total of 84 children, ranging from two to 18 years of age, were enrolled in this study. Of these, 28 had isolated aortic stenosis, 32 had isolated pulmonary stenosis and 24 were healthy. Children with aortic and pulmonary stenosis associated with other congenital heart diseases were excluded. Children with hypothyroidism, renal or liver disease, malignancy or autoimmune disease were also excluded. Wholeblood count, blood group, factor VIII level, prothrombin time (PT), activated partial thromboplastin time (aPTT), von Willebrand factor antigen (VWF:Ag), ristocetin co-factor (VWF:RCo), and bleeding time using a platelet-function analyser (PFA-100) were performed in all patients. All of the children in the study underwent a detailed physical examination and echocardiographic evaluation.

**Results:**

A history of bleeding was positive in 18% of the aortic stenosis group, 9% of the pulmonary stenosis group, and 4% of the control group. Seven of 60 (12%) patients had laboratory findings that implied a diagnosis of AVWS, and two of these (28%) had a history of bleeding. The frequency of AVWS was 14% in patients with aortic stenosis and 9% in those with pulmonary stenosis.

**Conclusion:**

AVWS is not rare in stenotic obstructive cardiac diseases. A detailed history of bleeding should be taken from patients with valvular disease. Even if the history is negative, whole blood count, PT and aPTT should be performed. If necessary, PFA-100 closure time and further tests should be planned for the diagnosis of AVWS.

Acquired von Willebrand syndrome (AVWS) is a rare clinical condition characterised by prolonged bleeding time and decreased levels of factor VIII and von Willebrand factor, as in congenital von Willebrand disease (VWD). It was first defined by Simone et al. in a seven-year-old boy with systemic lupus erythematosus.^1^ The actual prevalence of AVWS is uncertain. From 1968 to 1999, a total of 266 cases were reported. The International Society of Thrombosis and Haemostasis reported a retrospective analysis of 186 cases in 2000.^2^

AVWS is usually associated with underlying diseases such as lymphoproliferative disorders (48%), cardiovascular diseases (21%), myeloproliferative disorders (15%), other neoplasms (5%) and autoimmune diseases (2%).^2^ In rare cases, AVWS is also associated with hypothyroidism, uraemia and certain drugs, such as valproic acid and ciprofloxacin.

A few studies have reported on the association between AVWS and aortic stenosis in adults, but there are not enough studies reporting on the relationship between AVWS and right ventricular outflow obstruction. In patients with aortic stenosis, there is a loss of high-molecular-weight multimers, which are active in coagulation, due to increased shear stress. This situation can cause bleeding problems after menstruation, circumcision, dental extraction and congenital heart disease surgery.

No other coagulation tests are routinely used in people with heart disease, because AVWS has been identified in only a small number of patients. Therefore, it is of importance to identify AVWS in children with congenital heart diseases.^1^,^3^-^5^ In this study, we aimed to evaluate AVWS in children with aortic and pulmonary stenosis, and their clinical consequences.

## Methods

A total of 84 children, ranging from two to 18 years of age, were enrolled in this study. Of these, 28 had isolated aortic stenosis, 32 had isolated pulmonary stenosis and 24 were healthy. Children with aortic and pulmonary stenosis associated with other congenital heart diseases were excluded. Children with hypothyroidism, renal or liver disease, malignancy or autoimmune disease were also excluded.

A standardised screening questionnaire was used to evaluate each patient’s bleeding symptoms. Written informed consent was obtained from the parents of each patient, and the local ethics committee approved the study. Drugs and foods that could affect the coagulation tests were stopped one week before the evaluation. All the children in the study underwent a detailed physical examination and echocardiographic evaluation.

Transthoracic echocardiography was performed with a Vivid 7 (GE Vingmed, Horten, Norway) echocardiograph. M-modes of two-dimensional images were obtained from the parasternal long-axis views. Interventricular septal wall thickness, left ventricular posterior wall thickness and left ventricular internal diameters were measured in all the children. Cardiac chamber sizes and left ventricular systolic and diastolic function were assessed in accordance with the guidelines of the American Society of Echocardiography.6 The mean and peak transvalvular pressure gradients were calculated using the modified Bernoulli equation.

Patients with aortic stenosis were classified according to peak pressure gradient as insignificant (< 25 mmHg), mild (25–50 mmHg), moderate (50–74 mmHg) or severe (> 75 mmHg).^7^ Patients with pulmonary stenosis were classified according to peak gradient as mild (< 36 mmHg), moderate (36–64 mmHg) and severe (> 64 mmHg).^8^,^9^

The following blood collection and laboratory assays were performed: whole blood count, blood group, factor VIII level, prothrombin time (PT), activated partial thromboplastin time (aPTT), von Willebrand factor antigen (VWF:Ag), ristocetin co-factor (VWF:RCo), bleeding time using a platelet-function analyser (PFA-100; Dade Behring, Marburg, Germany) and platelet aggregation using a lumi-aggregometer.

The PFA-100 is a high-shear system for *in vitro* testing of platelet function that simulates primary haemostasis after injury to a small vessel by determining the closure time of adenosine diphosphate (ADP) cartridges. It is a highly sensitive way to screen patients for von Willebrand factor defect.^10^,^11^

Plasma VWF:Ag levels were evaluated by immunoturbidometry. VWF:Ag and VWF:RCo levels were standardised according to the blood group of each patient. Platelet aggregation tests were studied in 14 patients in whom the PFA-100 ADP collagen closure time was prolonged by low levels of VWF:Ag and VWF:RCo.

A diagnosis of AVWS was established by the following: (1) acquired history of bleeding; (2) low values of VWF:RCo and collagen-binding capacity (VWF:CB); and (3) VWF:RCo/ VWF:Ag and VWF:CB/VWF:Ag ratios less than 0.7 in cases of borderline or normal values of VWF:RCo and VWF:CB, and no curves in the platelet-aggregation test with ristocetin (RIPA).^2^

The gold standard for the detection of structural abnormalities of VWF is multimer analysis using electrophoretic separation and immunostaining. However this labour-intensive and time-consuming assay is not available in many laboratatories. Interpretation of the von Willebrand profile analysis is summarised in Table 1.

**Table 1:** Abbreviations and interpretations in the study

*Test name*	*Abbreviation*	*Interpretation*
Von Willebrand factor antigen	VWF:Ag	Measurement of the quantity of VWF monomers but no information given about its functional ability
Ristocetin co-factor assay	VWF:Rco	Measurement of the ability of VWF to agglutinate formalin-fixed platelets in presence of ristocetin
Ristocetin-to-VWF antigen ratio	VWF:Rco/VWF:Ag	Parameter of the capacity of available VWF to bind platelets
Collagen-binding capacity	VWF:CB	Measurement of the ability of high molecular weight VWF multimers to bind to sub-endothelial collagen
Collagen-binding capacity-to-VWF antigen ratio	VWF:CB/VWF:Ag	Measurement of the biological capacity of available VWF for binding to collagen.
Platelet-functional analyser (PFA-100) closure time (collagen and epinephrine) or (collagen and ADP)	PFA-100 CEPI or PFA-100 CADP	Screening test for primary haemostasis. Evaluates platelet disorders and functions.
Ristocetin-induced platelet aggregation	RIPA	Measurement of the ability of various agonists to platelets to aggravate *in vitro* activation and platelet-to-platelet activation

## Statistical analysis

SPSS software version 13.0 (SPSS Inc, Chicago, IL) was used for analysis. All results were expressed as mean ± SD. Assessment of significance between the groups was evaluated with the chi-squared and Mann–Whitney U-test and one-way ANOVA. Correlations between variables were assessed with Pearson’s rank-correlation test. A p-value < 0.05 was considered significant.

## Results

The study group consisted of 28 patients with aortic stenosis (23 males, five females), 32 patients with pulmonary stenosis (18 males, 14 females), and 24 healthy children (14 males, 10 females). The mean ages were 8.09 ± 3.73 years in the aortic stenosis group, 5.72 ± 3.79 years in the pulmonary stenosis group, and 9.12 ± 4.70 years in the control group.

A history of bleeding was positive in five patients with aortic stenosis, three with pulmonary stenosis and in one healthy child (Table 2). The mean follow-up time in patients with aortic stenosis was 4.19 ± 1.91 years, and 3.28 ± 1.98 years in patients with pulmonary stenosis (Table 3).

**Table 2:** Episodes of bleeding of study group

*Bleeding type*	*Patients (n)*	*Diagnosis*
Bleeding after circumcision	2	1 AS, 1 PS
Epistaxis	3	1 healthy, 2 AS
Bleeding after minor trauma	2	1 AS, 1 PS
Bleeding after dental extraction	1	AS
Postoperative bleeding	1	PS

AS: aortic stenosis, PS: pulmonary stenosis.

**Table 3:** Demographic features of the study groups

	*Aortic stenosis (n = 28)*	*Pulmonary stenosis (n = 32)*	*Control (n = 24)*	*p-value*
Gender (M/F)	23/5	18/14	14/10	0.07
Age (year)	8.09 ± 3.73	5.72 ± 3.79	9.12 ± 4.70	0.007
Follow-up period (year)	4.19 ± 1.91	3.28 ± 1.98	-	0.07
Aortic PPG (mmHg)	46.84 ± 17.63*	13.24 ± 2.78	10.71 ± 2.34	< 0.001
Aortic MPG (mmHg)	24.43 ± 10.59	-	-	0.004
Pulmonary PPG (mmHg)	12.7 ± 1.49	47.15 ± 11.70**	11.26 ± 2.18	< 0.001
Pulmonary MPG (mmHg)	-	24.95 ± 7.65	-	0.003

M: male, F: female, MPG: mean pressure gradient, PPG: peak pressure gradient.

*p < 0.001 aortic stenosis versus pulmonary stenosis and control.

**p < 0.001 pulmonary stenosis versus aortic stenosis and control.

Echocardiographic findings: the distribution of patients grouped by degree of stenosis is provided in Table 4.

**Table 4:** The distribution of patients according to degree of stenosis

*Group*	*Mild*	*Moderate*	*Severe*	*Total*
Aortic stenosis, n (%)	19 (67.9)	6 (21.4)	3 (10.7)	28 (46.7)
Pulmonary stenosis, n (%)	3 (9.4)	25 (78.1)	4 (12.5)	32 (53.3)
Total, n (%)	22 (36.7)	31 (51.7)	7 (11.7)	60 (100)

Haematological parameters: although the PT was in the normal range in patients with aortic and pulmonary stenosis, their PT values were found to be significantly shorter than that of the control group (p = 0.03 and 0.006, respectively). There was no significant difference between the PTs of the aortic and pulmonary stenosis groups (p = 0.52). The aPTT of the patients with pulmonary stenosis was significantly higher than that of the control group (p = 0.019) (Table 5).

**Table 5:** Comparison of haematological parameters of the groups

*Haematological parameters*	*Aortic stenosis (n = 28)*	*Pulmonary stenosis (n = 32)*	*Control (n = 24)*	*p-value*
Platelet count (cells/mm³)	336.570 ± 58.053	356.440 ± 103.244	346.920 ± 58.670	0.62
PT (s)	13.46 ± 0.64	13.34 ± 0.68	13.87 ± 0.75^a^	0.017
aPTT (s)	29.55 ± 2.06	30.49 ± 3.48^b^	28.82 ± 1.50	0.059
Factor VIII (%)	123.57 ± 42.92	118.69 ± 47.71	130.79 ± 37.35	0.58
VWF:Ag (%)	100.86 ± 31.97	98.44 ± 31.34	97.42 ± 21.49	0.90
VWF:RCo (%)	94.75 ± 29.36	97.28 ± 34.28	89.00 ± 19.04	0.56
PFA-100 CADP (s)	116.86 ± 45.14	117.34 ± 46.75	-	0.96
PFA-100 CEPI (s)	178.64 ± 56.19	168.22 ± 55.44	-	0.47
VWF:RCo/VWF:Ag	0.95 ± 0.21	1.01 ± 0.35	0.91 ± 0.12	0.36

PT: prothrombin time; aPTT: activated partial thromboplastin time; PFA-100 CADP: platelet-function analyser-100 adenosine diphosphate collagen closure time; PFA-100 CEPI: platelet-function analyser-100 collagen epinephrine closure time; VWF:Ag: von Willebrand factor antigen; VWF:RCo: ristocetin co-factor. PFA-100 ADP closure time was carried out in only patients with aortic and pulmonary stenosis (n = 60), but the other tests were done in all children (n = 84).

^a^p = 0.03, aortic stenosis versus control group and p = 0.006, pulmonary stenosis versus control group.

^b^p = 0.019, pulmonary stenosis versus control group.

**Fig. 1.**
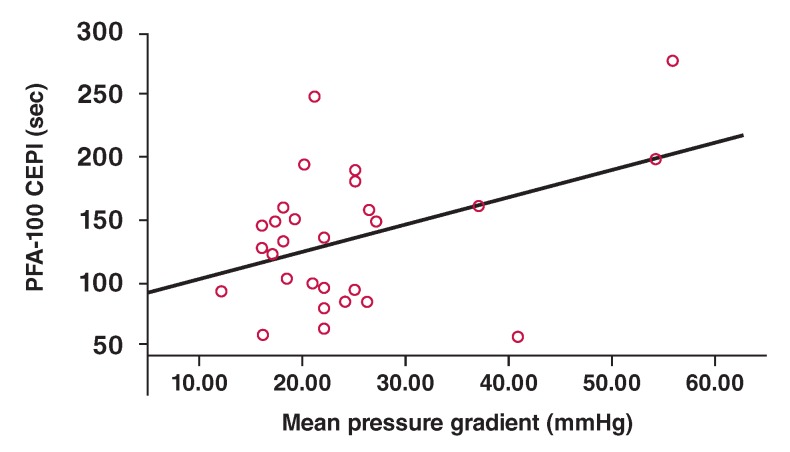
Correlation between PFA-100 collagen epinephrine closure time and mean pressure gradient in patientswith aortic stenosis.

The VWF:Ag and VWF:RCo levels were not statistically different among the patients with aortic stenosis according to degree of stenosis (Table 6). PFA-100 collagen epinephrine closure time was significantly higher in patients with severe aortic stenosis than in those with mild or moderate aortic stenosis (p = 0.003 and p = 0.01, respectively). PFA-100 collagen epinephrine closure times were positively correlated with degree of aortic stenosis (p = 0.03) ((Fig. 1)).

**Table 6:** The comparison of haematological parameters of patients with aortic stenosis according to degree of stenosis

**	*Mild aortic stenosis (n = 19) (mean ± SD)*	*Moderate aortic stenosis (n = 6) (mean ± SD)*	*Severe aortic stenosis (n = 3) (mean ± SD)*	*p-value*
Platelets (cells/mm³)	329.680 ± 57.915	352.000 ± 60.332	349.330 ± 69.292	0.67
PT (s)	13.579 ± 0.642	13.183 ± 0.752	13.267 ± 0.208	0.37
aPTT (s)	30.005 ± 1.658	28.300 ± 3.039	29.167 ± 1.656	0.20
Factor VIII (%)	120.21 ± 39.76	132.67 ± 63.96	126.67 ± 6.02	0.82
VWF:Ag (%)	101.32 ± 35.17	95.17 ± 27.27	109.33 ± 24.50	0.82
VWF:RCo (%)	95.11 ± 29.37	98.00 ± 29.67	86.00 ± 39.05	0.85
PFA-100 CADP (s)	110.74 ± 39.79	119.00 ± 32.33	151.33 ± 92.13	0.36
PFA-100 CEPI (s)	166.37 ± 52.05	173.50 ± 34.51	266.67 ± 46.14^c^	0.01
VWF:RCo/vVWF:Ag	0.95 ± 0.19	1.03 ± 0.27	0.76 ± 0.20	0.21

PT: prothrombin time; aPTT: activated partial thromboplastin time; PFA-100 CADP: platelet-function analyser-100 adenosine diphosphate collagen closure time; PFA-100 CEPI: platelet-function analyser-100 collagen epinephrine closure time; VWF:Ag: von Willebrand factor antigen; VWF:RCo: ristocetin co-factor.

^c^p = 0.003, severe versus mild aortic stenosis, p = 0.01, severe versus moderate aortic stenosis.

The platelet count of patients with mild pulmonary stenosis was significantly lower than that of patients with severe pulmonary stenosis (p = 0.04). The PT of the patients with severe pulmonary stenosis was significantly higher than that of those with moderate pulmonary stenosis (p = 0.003). The aPTT of the patients with mild pulmonary stenosis was significantly higher than that of those with moderate and severe pulmonary stenosis (p = 0.002 and p = 0.009, respectively) (Table 7). PFA-100 collagen epinephrine and PFA-100 ADP closure times were not different among the patients with pulmonary stenosis (p = 0.93 and p = 0.18).

**Table 7:** The comparison of haematological parameters of patients with pulmonary stenosis according to degree of stenosis

	*Mild pulmonary stenosis (n = 3) (mean ± SD)*	*Moderate pulmonary stenosis (n = 25) (mean ± SD)*	*Severe pulmonary stenosis (n = 4) (mean ± SD)*	*p-value*
Platelet (cells/mm³)	308.330 ± 52.596^d^	345.800 ± 97.395	459.000 ± 123.018	0.01
PT (s)	13.667 ± 0.152	13.204 ± 0.649^e^	14.000 ± 0.812	0.05
aPTT (s)	36.267 ± 7.691^f^	29.920 ± 2.186	29.775 ± 3.313	0.007
Factor VIII (%)	93.33 ± 21.07	116.80 ± 44.65	149.50 ± 73.07	0.28
VWF:Ag (%)	91.67 ± 28.00	95.88 ± 28.72	119.50 ± 48.37	0.35
VWF:RCo (%)	115.00 ± 22.71	93.88 ± 33.86	105.25 ± 46.21	0.54
PFA-100 CADP (s)	163.00 ± 98.73	110.72 ± 39.85	124.50 ± 29.49	0.18
PFA-100 CEPI (s)	157.00 ± 37.16	169.64 ± 54.78	167.75 ± 81.80	0.93
VWF:RCo/ VWF:Ag	1.33 ± 0.57	0.99 ± 0.33	0.87 ± 0.14	0.20

PT: prothrombin time; aPTT: activated partial thromboplastin time; PFA-100 CADP: platelet-function analyser-100 adenosine diphosphate collagen closure time; PFA-100 CEPI: platelet-function analyser-100 collagen epinephrine closure time; VWFAg: von Willebrand factor antigen; VWF:RCo: ristocetin co-factor.

^d^p = 0.04, severe versus mild pulmonary stenosis.

^e^p = 0.003, severe versus moderate pulmonary stenosis.

^f^p = 0.002, mild versus moderate pulmonary stenosis, p = 0.009, mild versussevere pulmonary stenosis.

VWF:Ag levels were normal in all patients with aortic stenosis and in the control group. However, it was low in three patients with pulmonary stenosis (two female, one male) and all were asymptomatic. The family histories of these three patients were negative for bleeding. VWF:RCo was lower in one patient who had a deficiency of VWF:Ag. Of the patients with low VWF:Ag, PFA-100 ADP collagen closure time was prolonged in two patients, and PFA-100 collagen epinephrine closure time was prolonged in all of them. PFA-100 ADP collagen closure time was prolonged in three patients with aortic stenosis and PFA-100 collagen epinephrine closure time was prolonged in four patients with aortic stenosis. Only two of four patients with prolonged PFA-100 collagen epinephrine closure time had a positive bleeding history (Table 8)

**Table 8:** The clinical and laboratory features of patients with abnormal platelet aggregation tests and low VWF:RCo/VWF:Ag

*Age*	*Diagnosis*	*Gender*	*Symptoms*	*Blood group*	*VWF: Ag (%)*	*VWF: Rco (%)*	*PFA-100 CADP*	*PFA-100 CEPI*	*VWF:RCo/ VWF:Ag*	*PPG (mmHg)*	*MPG (mmHg)*
18	PS	F	No	B RH+	55 (↓)	99 (N)	100 (N)	179 (↑)	1.8 (N)	49	25
3	PS	M	No	AB RH+	52 (↓)	46 (↓)	135 (↑)	263 (↑)	0.88 (N)	42	20
7	PS	F	No	AB RH+	64 (↓)	131 (N)	277 (↑)	196 (↑)	2 (N)	30	12
5	AS	M	Yes	O RH+	56 (N)	49 (↓)	115 (N)	212 (↑)	0.87 (N)	36	18
10	AS	F	No	A RH+	62 (N)	49 (↓)	210 (↑)	182 (↑)	0.79 (N)	40	21
6	AS	M	No	O RH+	58 (N)	46 (↓)	155 (↑)	185 (↑)	0.79 (N)	56	25
8*	AS	F	Yes	O RH+	75 (N)	53 (N)	137 (↑)	217 (↑)	0.7 (N)	34	18

AS: aortic stenosis; F: female; M: male; MPG: mean pressure gradient; N: normal; PS: pulmonary stenosis; PFA-100 CADP: platelet-function analyser-100 adenosine diphosphate collagen closure time; PFA-100 CEPI: platelet-function analyser-100 collagen epinephrine closure time; VWF:Ag: von Willebrand factor antigen; VWF:RCo: ristocetin co-factor; PPG: peak pressure gradient.

*This patient’s platelet aggregation curve induced by ristocetin was horizontal.

PFA-100 ADP collagen closure times and PFA-100 collagen epinephrine closure times were performed only in patients with aortic and pulmonary stenosis. PFA-100 ADP collagen closure time was normal in 40 (66.7%) patients and prolonged in 20 (33.3%). Of the 20 patients, 11 had aortic stenosis (six had mild stenosis, three had moderate stenosis and two had severe stenosis), and nine had pulmonary stenosis (one mild, six moderate and two severe).

PFA-100 collagen epinephrine closure time was prolonged in 34 (56.6%) patients. Of these, 19 had aortic stenosis (12 mild,four moderate, three severe) and 15 had pulmonary stenosis (one mild, 12 moderate, two severe). A total of 16 of 60 patients who underwent PFA-100 ADP collagen closure time and PFA-100 collagen epinephrine closure time evaluation had a positive bleeding story (26.6%) (Table 9, Table 10).

**Table 9:** Abnormal haematological parameters between the study groups

**	*Aortic stenosis (n = 28)*	*Pulmonary stenosis (n = 32)*	*p-value*
Low VWF:Ag, n (%)	0	3 (100)	0.08
Low VWF:RCo, n (%)	3 (75)	1 (25)	0.16
Prolonged PFA-100 CADP, n (%)	11 (55)	9 (45)	0.36
Prolonged PFA-100 CEPI, n (%)	19 (56)	15 (44)	0.10
Low VWF:RCo/VWF:Ag, n (%)	1 (50)	1 (50)	0.66

VWF:Ag: von Willebrand factor antigen; VWF:RCo: ristocetin co-factor; PFA-100 CADP: platelet-function analyser-100 adenosine diphosphate collagen closure time; PFA-100 CEPI: platelet-function analyser-100 collagen epinephrine closure time.

**Table 10:** Relationship between VWF components and PFA-100 CEPI and CADP

	*PFA-100 CADP*			*PFA-100 CEPI*		
	*Normal (n = 40)*	*Prolonged (n = 20)*	*p-value*	*Normal (n = 26)*	*Prolonged (n = 34)*	*p-value*
Low VWF:Ag	1	2	0.20	–	3	0.12
Low VWF:RCo	1	3	0.06	–	4	0.07
Positive bleeding history	3	5	0.06	2	6	0.26

VWF:Ag: von Willebrand factor antigen; VWF:RCo: ristocetin co-factor; PFA-100 CADP: platelet-function analyser-100 adenosine diphosphate collagen closure time; PFA-100 CEPI: platelet-function analyser-100 collagen epinephrine closure time.

## Discussion

VWD is classified into three main types. Type 1 VWD is the most common form of disorder and is characterised by a mildto- moderate decrease in the plasma levels of VWF. Plasma VWF from these individuals has a normal structure. Plasma levels ofristocetin co-factor and factor VIII tend to be proportionately decreased.

Type 2A VWD is associated with the absence of large highmolecular- weight (HMW) multimers from the plasma and platelets, which results in impaired ability to mediate platelet adhesion to the endothelium. This type is common in patients with cardiac stenotic diseases, especially in aortic stenosis, because of mechanical destruction of the HMW multimers of VWF under high shear stress.

Type 2B VWD is a rare form of VWD characterised by partial loss of plasma HMW multimers and increased responsiveness when exposed *ex vivo* to the antibiotic ristocetin. In Type 3 VWD, plasma levels of factor VIII and VWF are virtually undetectable.^12^

Studies to investigate the prevalence of VWD in patients with heart disease are mainly carried out in adults. Although there are few reports in children, the relationship between congenital heart defects and VWD has been elucidated. In a study by Arslan et al., the prevalence of VWD was reported as 12.2% in 49 children with congenital heart disease.^13^ We found a similar ratio in our study (11.6%).

In a study by Gill et al.,^14^ abnormal von Willebrand factor multimers was detected in all patients with congenital heart disease, but only six of them had low VWF:Ag levels. In this study, multimer analysis was repeated on five patients after surgical correction, and four patients had a normal multimeric structure. Rauch et al. reported four cases of VWD in 12 patients with patent ductus arteriosus, and six months after closure of the defect, the abnormal levels of von Willebrand factor had returned to within normal limits.^15^

The prevalence of bleeding episodes in patients with congenital heart disease is controversial. Several studies reported that at least 20% of patients with aortic stenosis have a history of bleeding.^16^-^18^ In the study by Gill et al.,^14^ 66% of patients with ventricular septal defect and 50% with aortic stenosis had bleeding episodes; however, patients with atrial septal defect did not have bleeding episodes. Most of these episodes were excessive bleeding after injury or epistaxis, and easy bruising.

In our study, two of seven patients (28.5%) with a diagnosis of AVWS had episodes of bleeding. These two patients had aortic stenosis, and PFA-100 collagen epinephrine closure time was prolonged in both.

Although Froom et al.^19^ reported a relationship between frequent epistaxis episodes and low VWF:Ag, Rauch et al.^15^ did not find any association between bleeding episodes and the von Willebrand deficiency in patients with patent ductus arteriosus. We also could not find any relationship between low VWF:Ag levels and bleeding. In our study, it was noteworthy that 33% of patients with pulmonary stenosis with prolonged PFA-100 ADP collagen closure times had bleeding episodes.

The PFA-100 closure time has been suggested to be useful in screening for disorders of primary haemostasis, including VWD.^9^ In a study by Vincentelli et al.,^17^ 92% of patients with severe aortic stenosis and 50% of patients with moderate aortic stenosis had prolonged PFA-100 ADP collagen closure times. However, in our study, 67% of the patients with severe aortic stenosis and 50% of those with moderate aortic stenosis had prolonged PFA-100 ADP collagen closure times.

In our study, PFA-100 collagen epinephrine closure time was prolonged in all seven patients with impaired haematological parameters matching AVWS. Only one of seven patients had a bleeding history, which suggests that absence of a history of bleeding episodes is not enough to exclude AVWS. For this reason, patients with heart disease should be screened with PFA-100 closure time before any intervention or surgery, even in the absence of a history of bleeding.

Yoshida et al.^16^ reported a positive correlation between VWF:Ag levels and effective valvular area in patients with aortic stenosis. However, we were unable to show any correlation between degree of valvular stenosis and VWF:Ag and VWF:RCo levels.

Routine coagulation tests and thrombo-elastometric measurements are not enough to exclude a diagnosis of AVWS. Laboratory investigations for AVWS include patient’s history of bleeding (lifelong mucocutaneous, postoperative bleeding) and a family history of bleeding, screening procedures [e.g. platelet count, PTT, concentration of factor VIII (FVIII:C), PFA-100], and confirming tests [e.g. VWF:Ag, VWF:RCo, VWF collagenbinding activity (VWF:CB), RIPA, and analysis of VWF multimers by gel electrophoresis].^20^ Most of these tests are time consuming and available only in specific centres.

In some studies, the VWF:Ag levels were normal, while in others, they were low.^13^, ^15^,^17^,^20^ In our study, VWF:Ag levels were normal in all the healthy children and in the patients with aortic stenosis, but they were low in 5% of patients with pulmonary stenosis. The VWFR:RCo level was low in one of three patients with low VWF:Ag levels. In a study by Gill et al.,^14^ VWF:RCo levels were low in seven of 12 patients with acyanotic congenital heart disease. Only 6% of our patients had low VWF:Ag levels.

Early diagnosis of AVWS is difficult, due to a lack of sensitivity of the tests used. Tiede et al.^5^ found that the sensitivity of PFA-100 was 80% for the diagnosis of AVWS. In our study, the sensitivity of VWF:Ag (23%) and VWF:RCo/VWF:Ag < 0.7 (26%) was too low to rule out this disease. We agree with Tiede et al.^5^ They suggested that a substantial number of patients present with normal or abnormal test results. The analysis of VWF multimers should always be part of the diagnostic workup.^5^

There were some study limitations; first, the low number of patients in each group, and second, for financial reasons, we could not perform VWF multimer analysis, which is the gold-standard test for the diagnosis of acquired VWD. Finally, patients in the control group did not undergo PFA-100 closure time tests.

## Conclusion

We suggest that a history of bleeding must be evaluated carefully in patients with stenotic obstructive heart disease, due to the increased risk of AVWS. Furthermore, even in the absence of bleeding episodes, PFA-100 closure time should be evaluated routinely before any intervention or surgery. If any abnormality is detected, VWF:Ag, VWF:RCo and platelet aggregation tests should be performed. Although the gold-standard test for AVWS is detection of structural abnormalities of VWF, it is a time-consuming assay and not available in most centres.
